# Crossed Fused Renal Ectopia With Acute Pyelonephritis: A Case Report

**DOI:** 10.7759/cureus.19907

**Published:** 2021-11-25

**Authors:** Praraj Jaiswal, Dhruv Talwar, Sourya Acharya, Samarth Shukla, Sunil Kumar

**Affiliations:** 1 Department of Medicine, Jawaharlal Nehru Medical College, Datta Meghe Institute of Medical Sciences, Wardha, IND; 2 Department of Pathology, Jawaharlal Nehru Medical College, Datta Meghe Institute of Medical Sciences, Wardha, IND

**Keywords:** congenital anomaly, nephrology, ectopic kidney, acute pyelonephritis, crossed fused renal ectopia

## Abstract

Crossed Fused Renal Ectopia (CFRE) is a very rare anomaly of the kidneys with a prevalence of 1 in 1000 live births. In this condition, both kidneys are fused together on one side of the spine. Usually, CFRE is an asymptomatic condition and is mostly detected incidentally, but sometimes the patient may develop various renal signs and symptoms. We report a case of acute pyelonephritis in a crossed fused right renal ectopia which was diagnosed by renal ultrasound and confirmed by computed tomography in a 75-year-old female.

## Introduction

Ectopic kidney is a congenital condition in which one or both kidneys are located in an incorrect position. The incidence of crossed renal ectopia is believed to be around 1 in 1000 newborns. There is a clear masculine preference, with a male to female ratio of 2:1. Ectopic kidneys come in a variety of shapes and sizes (e.g., pelvic kidney, thoracic kidney, and crossed fused renal ectopia [CFRE]). CFRE or crossed dystopia is an uncommon type of renal ectopia in which both kidneys lie on the same side of the spine [1,2]. Pyelonephritis in CFRE patients is quite uncommon.

## Case presentation

 A 75-year-old female patient presented to us with right-sided flank pain, low-grade fever with chills, and burning micturition for ten days. There was no history of cough, hematuria, vomiting, or diarrhea. On examination, the temperature was 1010°F, pulse was 118/minute. The rest of the general examination was normal. Abdominal examination revealed tenderness in the right lumbar region and renal angle tenderness on the right side. She was non-diabetic and non-hypertensive.

A routine urine examination revealed plenty of pus and epithelial cells in the urine. Laboratory investigations are mentioned in Table 1.

**Table 1:** Showing Laboratory Investigations of the Case

Laboratory parameter	Measured Value
White Blood Cell Count	14200 per microliter
Neutrophils	82%
Haemoglobin	12.4gm/dl
Platelet count	467000 per microliter
Serum Urea	87 mg/dl
Serum Creatinine	2.2 mg/dl
Serum Sodium	142 mEq/L
Serum Potassium	5.2 mmol/l
Serum Calcium	9.5mg/dl
Alkaline phosphate	90 IU/L
Alanine Aminotransferase	14 U/L
Aspartate aminotransferase	35 U/L
Total protein	8.4 g/dl
Total bilirubin	0.6 mg/dl
C-Reactive Protein	50 milligram per litre
Erythrocyte Sedimentation Rate	42 millimeter per hour

**Figure 1:**
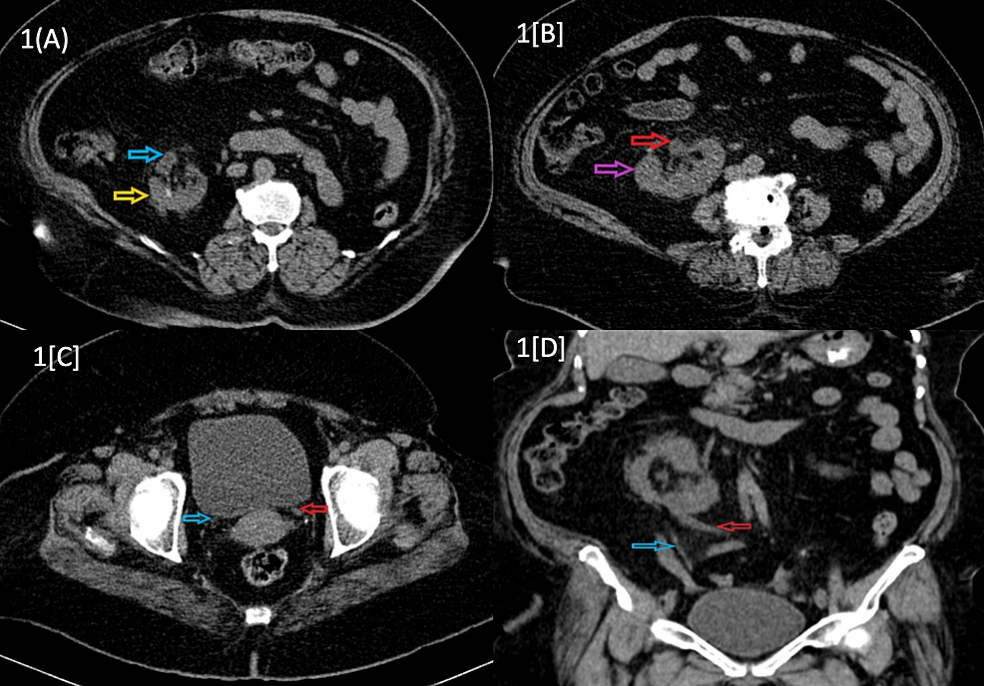
(A) CT axial section showing right kidney in right renal fossa (yellow arrow) with pelvis rotated anteriorly (blue arrow). (B) CT axial section showing left kidney in the lumbar region (purple arrow) with pelvis rotated anterolateral (red arrow). (C) CT axial section showing left ureter (red arrow) and right ureter (blue arrow) normal opening in the trigone of the bladder. (D) CT coronal section showing left ureter (red arrow) crossing midline and right ureter (blue arrow) is following its normal course

Abdominal ultrasonography was performed, which was suggestive of grossed fused ectopia on the right side with mildly raised echotexture of kidneys. CT abdomen was performed to confirm the diagnosis, which indicated normal size and position of right kidney but left kidney located in the right lumbar region with the fusion of upper pole of the left kidney at the lower pole of right kidney suggesting crossed fused renal ectopia with left kidney on the right side and normal opening of the left ureter (Figure 1). There was also finding of perinephric and periureteric fat stranding suggestive of pyelonephritis. Renal colour Doppler was performed to view the vascularity of both kidneys, which revealed right renal fossa shows crossed fused ectopic kidney measures 16.3 x 6.4 cm. An increased resistive index of 0.9 was noted in segmental arteries. A mild tardus parvus pattern was noted.

A urine culture was done, which showed growth of extended-spectrum β-lactamases [ESBL] producing *Escherichia coli* species that was sensitive to carbapenems. The patient was treated with intravenous meropenem 1 gram bid for one week and hydration. The patient improved clinically after antibiotic therapy of seven days. Serum urea and creatinine were 30 mg/dl and 1.9 mg/dl at the time of discharge. On follow-up after two weeks of discharge patient was asymptomatic and urea and creatinine levels were normal. The patient is currently doing well on follow-up with negative urine culture and routine microscopy on day 15.

## Discussion

Crossed renal ectopia results in fusion in more than 90% of cases. As seen in Table 2, they are divided into six subgroups in decreasing order of frequency [3]. It is caused by aberrant renal ascent during embryogenesis, which leads to the fusion of the kidneys within the pelvis. It's thought to happen in the first trimester, during the fourth or eighth week of life for the foetus.

**Table 2:** Showing classification of crossed fused renal ectopia into six subtypes with decreasing order of frequency

Subtype	Location in abdomen
Type A	inferiorly crossed fusion
Type B	sigmoidal kidney
Type C	lump kidney
Type D	disc kidney
Type E	L-shaped kidney
Type F	superior crossed fused

Both kidneys are on the same side in this abnormality, as they are in our patient, with the majority of cases being fused and two separate ureters coming from the respective kidneys. The ureter from the crossed-over kidney, which is the left kidney, in our case returned to the opposite side and inserted in the bladder, whereas the ureter from the right kidney followed the regular path. A majority of these cases are discovered by chance because they are usually asymptomatic. If symptomatic, stomach or flank pain, palpable lump, dysuria, or haematuria are the most common presenting signs [3]. Ureteral orifices are normally located in the normal place, with ectopic ureteric orifices occurring in 3% of instances [3,4]. These defects can also be linked to conditions including vesicoureteric reflux, ureterocoele, nephrolithiasis, Ureteropelvic Junction blockage, and, in rare cases, cancer [3].

Ultrasound, intravenous urography, or anterograde pyelography, isotopic investigations, computed tomography, and magnetic resonance imaging are used to make the diagnosis. Imaging will continue to play a key role in the diagnosis of congenital renal disorders [4]. Because of its quick scanning time, lack of radiation, low cost, and ease of feasibility, ultrasound is frequently used for its detection and evaluation, and Doppler ultrasound aids in accurate diagnosis through the study of vascularization. This is beneficial because ectopic kidneys typically have a large number of tiny arteries that reflect the continual changes in blood supply as the kidneys develop until they reach their final position [5]. The only treatment for this congenital abnormality is to address the pathology affecting the kidney; if the patient is asymptomatic, no other treatment is indicated.

## Conclusions

Though a rare asymptomatic congenital condition, crossed fused renal ectopia may present with serious complications such as pyelonephritis and obstructive uropathy. Hence, early diagnosis of this congenital malformation is very important. One should rule out any likelihood of such abnormalities leading to obstruction, infection, or neoplasia of the urinary system later in life, not only in adulthood but also throughout the antenatal period.
